# Bizarre wide complex tachycardia caused by sodium channel toxicity secondary to the management of status epilepticus: Case report

**DOI:** 10.1016/j.amsu.2022.104558

**Published:** 2022-09-02

**Authors:** Ghazi T. AlMutairi

**Affiliations:** Department of Medicine, Unaizah College of Medicine and Medical Science, Qassim University, Saudi Arabia

**Keywords:** Case report, Antiepileptic drugs (AED), Sodium channels blockade, Wide complex arrhythmias, Extracorporeal removal

## Abstract

**Introduction and importance:**

Antiepileptic drugs therapy are associated with myriad adverse effects. Some of antiepileptic drugs (AEDs) inhibit sodium channels that may cause dysrhythmia and hemodynamic instability if administered in too high dose.

**Case presentation:**

We describe a patient with wide complex tachycardia likely secondary to medical management of status epilepticus specifically secondary to sodium channels blockade that successfully managed with supportive care and one session of hemodialysis.

**Conclusion:**

In this case report, we discuss the successful management of the first reported combination of AEDs (Phenytoin and lacosamide) toxicity using supportive care and extracorporeal removal.

## Highlights

• Phenytoin and Lacosamide are sodium channels blockade.
• Sodium channels blockade toxicity can lead to lethal ventricular arrhythmias and hemodynamic instability.
• Extracorporeal removal of phenytoin and lacosamide can restore patient's rhythm and hemodynamic stability.

## 1 Introduction

Antiepileptic drugs (AEDs) most commonly inhibit sodium voltage-dependent channels that located on the cell membrane which are found on both neuronal and cardiac tissue. Phenytoin and lacosamide are AEDs which act through blockade of voltage-dependent sodium channels.

Sodium channels are important ion channels that responsible for transcellular sodium influx in the cardiac tissue [1]. Inhibition of cardiac sodium channels will manifest on the electrocardiogram (ECG) as prolongation of the QRS interval, deep S wave in lead AVL, right axis deviation, tall R wave and Brugada like pattern [2,3]. In cases of excessive administration of sodium channels blockade may lead to cardiac arrhythmias and hemodynamic instability [4].

The management approach in the setting of sodium channels blockade toxicity are similar to the tricyclic antidepressant toxicity management which includes discontinuation of offending agent, repeated doses of intravenous sodium bicarbonate with a target pH of 7.45–7.55 and continuous cardiac monitoring to assess the response to this intervention.

We present here a bizarre wide complex tachycardia secondary to sodium channels blockade toxicity that is refractory to standard management of such cases. In this case report, we discuss the successful management of the first reported sodium channels blockade toxicity using hemodialysis.

The case report has been reported in line with the SCARE 2020 criteria [5].

## 2 Case presentation

**Fig. 1:**
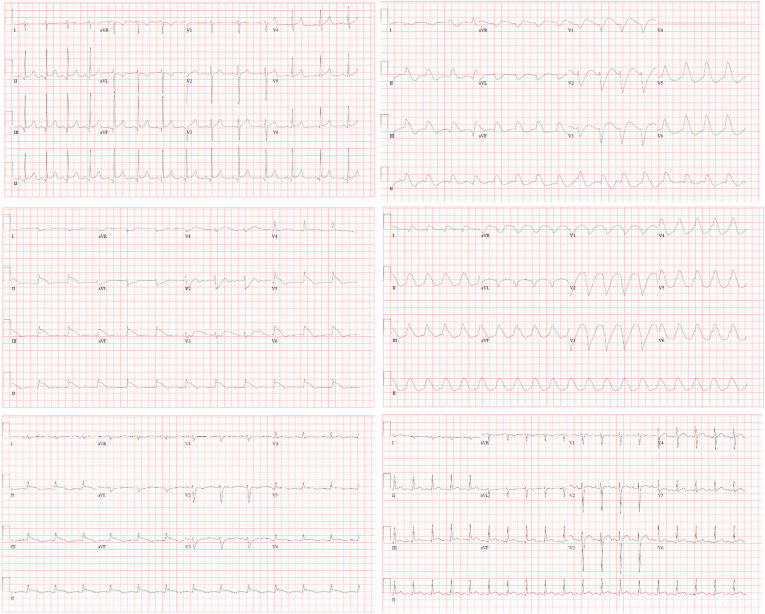
Progression of ECG changes over the span of the patient's admission to the ICU.

A 19-year-old female with a history of seizure disorder presented to the emergency department in status epilepticus. She had no prior history of cardiovascular disease or known family history suggestive of arrhythmia. She was unsuccessfully treated with lorazepam, then requiring escalation to propofol and endotracheal intubation. Enteral anti-epileptic therapy was started in conjunction with intravenous burst suppression using levetiracetam 1500mg BID, phenytoin 100mg TID, lacosamide 200mg BID and perampanel 6mg daily - titrated to EEG assessments over 48 hours. The patient also received a single dose of phenobarbital. The initials blood work up including complete blood counts, electrolytes, renal profile, hepatic profile and glucose were within the normal limits. On the third day of admission, the patient developed evidence of a wide complex tachycardia with a rate of 130bpm. Attempts to restore her rhythm with amiodarone, lidocaine, and electrical cardioversion has been failed. Careful assessment of her 12-lead ECGs revealed progressive widening of the QRS to 360 ms and prolongation of QTc to 640 ms via Bazett method (Fig. 1). The ECG changes suggest toxic effects of sodium channel blocking agents. Initial treatment included repeated doses of intravenous sodium bicarbonate followed by an infusion with a goal of pH 7.45–7.55, continuous cardiac monitoring, discontinuing amiodarone and lidocaine, as well as down-titrating the antiepileptic agents. Phenytoin and perampanel were discontinued and the lacosamide dose was reduced by 50%. Despite these changes, the patient developed a refractory wide complex tachycardia– as such urgent dialysis was initiated to increase the rate of drug elimination. Of note her total phenytoin level was 73 μmol/L (adjusted for albumin), at the upper limit of normal and we couldn't measure the free phenytoin level. Upon initiation of dialysis, the patient's QRS and QTc progressively narrowed to normal within 12 hours (Fig. 1). The patient's condition has been stabilized following the initial eight-hour run, and no further sessions were required.

## 3 Discussion

We present a case where combination of AEDs that acting as sodium channels blockers led to lethal cardiac arrhythmias and significant ECG changes similar to those seen in tricyclic antidepressant toxicity.

One potential mechanistic explanation to our case is phenytoin toxicity. While the total level is at the ULN, a free phenytoin level was not ascertained. Drug-drug interaction with protein-binding displacement and alternation in cytochrome p450 activity from concurrent administration of other anti-epileptics, including perampanel, may result in an elevated free phenytoin level, discordant with the measured total level. The other explanation to our case is a combination of two AEDs which acts through blockage of sodium channels led to paradoxical significant cardiac conduction abnormalities that might predispose to serious ventricular arrhythmias.

In this case report, treatment was directly aimed at resuscitation and stabilization followed by empiric hemodialysis to facilitate drugs elimination as our concerns were related to cardiac toxicity mainly ventricular arrhythmias and hemodynamic instability.

There are some data regarding ventricular arrhythmias and AEDs in the literature. Nizam A et al. Reported a case of conduction dysrhythmia induced by lacosamide [6]. Another case report by DeGiorgio et al. Reported as a case of ventricular arrhythmias related to lacosamide treatment [7].

The literature suggests considering extracorporeal removal in the sitting of severe carbamazepine toxicity and phenytoin toxicity in selected cases of severe poisoning but no data available for lacosamide [8,9]. We believe this is a reasonable approach after all standard management has been failed. We recommend consultation with a nephrologist and toxicologist if possible when hemodialysis is considered.

## 4 Conclusion

This case report describes toxicity associated with a combination of AEDs that caused significant ECG changes and life threatening arrhythmias and was successfully managed with supportive care and one session of hemodialysis that resulted in restoration of patient's condition and stability.

## Source of funding

No funding to be declared.

## Ethical approval

None.

## Consent

Written informed consent was obtained from the patient for publication of this case report and accompanying images. A copy of the written consent is available for review by the Editor-in-Chief of this journal on request.

## Author contribution

Ghazi T. AlMutairi: written the paper and treating physician.

## Research registration

None.

## Guarantor

Ghazi T. AlMutairi.

## Provenance and peer review

Not commissioned, externally peer reviewed.

## Declaration of competing interest

None.
